# The Effect of 10% Carbamide Peroxide Dental Bleaching on the Physical Properties of Invisalign Aligners: An In Vitro Study

**DOI:** 10.3390/ma16114125

**Published:** 2023-06-01

**Authors:** Majd Khashashneh, Jithendra Ratnayake, Joanne Jung Eun Choi, Li Mei, Karl Lyons, Paul Brunton

**Affiliations:** 1 Sir John Walsh Research Institute, Faculty of Dentistry, University of Otago, P.O. Box 56, Dunedin 9054, New Zealand; 2 Faculty of Dentistry, Jordan University of Science and Technology, P.O. Box 3030, Irbid 22110, Jordan; 3 DVCA, Curtin Perth, Curtin University, Bentley, WA 6102, Australia

**Keywords:** tooth whitening, dental bleaching, carbamide peroxide, Invisalign, tensile strength, hardness, mechanical properties

## Abstract

The high aesthetic demands of patients have increased their requests to align their teeth using clear aligners, including Invisalign. Patients also want to have their teeth whitened for the same purpose; the use of Invisalign as a bleaching tray at night has been reported in few studies. However, whether 10% carbamide peroxide affects the physical properties of Invisalign is unknown. Therefore, the objective of this study was to evaluate the effect of 10% carbamide peroxide on the physical properties of Invisalign when used as a bleaching tray at night. Twenty-two unused Invisalign aligners (Santa Clara, CA, USA) were used to prepare 144 specimens to test their tensile strength, hardness, surface roughness, and translucency. The specimens were divided into four groups: a testing group at baseline (TG1), a testing group after application of bleaching material at 37 °C for 2 weeks (TG2), a control group at baseline (CG1), and a control group after immersion in distilled water at 37 °C for 2 weeks (CG2). Statistical analysis was conducted using a paired t-test, Wilcoxon signed rank test, independent samples t-test, and Mann–Whitney test to compare samples in CG2 to CG1, TG2 to TG1, and TG2 to CG2. Statistical analysis showed no statistically significant difference between the groups for all physical properties, except for hardness (*p*-value < 0.001) and surface roughness (*p*-value = 0.007 and *p*-value < 0.001 for the internal and external surface roughness, respectively), which revealed a reduction in hardness values (from 4.43 ± 0.86 N/mm^2^ to 2.2 ± 0.29 N/mm^2^) and an increase in surface roughness (from 1.6 ± 0.32 Ra to 1.93 ± 0.28 Ra and from 0.58 ± 0.12 Ra to 0.68 ± 0.13 Ra for the internal and external surface roughness, respectively) after 2 weeks of dental bleaching. Results showed that Invisalign can be used for dental bleaching without excessive distortion or degradation of the aligner material. However, future clinical trials are required to further assess the feasibility of using Invisalign for dental bleaching.

## 1. Introduction

An increasing number of patients are seeking orthodontic treatment using aesthetic orthodontic appliances because media and websites have greatly influenced patients’ expectations and attitudes regarding dental treatment and aesthetics [1], leading to the emergence of clear aligners, including Invisalign [2], which was introduced by Align Technology (Santa Clara, CA, USA) in 1997 [3,4,5]. The advantages of Invisalign include aesthetics and convenience because the aligner can be removed for eating and tooth brushing [6,7]. Additional advantages of Invisalign include increased patient satisfaction compared to fixed orthodontic treatment, as reported in a number of studies [8,9,10].

The effectiveness of orthodontic treatment using Invisalign has been evaluated in the literature. One systematic review showed that Invisalign is a viable option for treating mild–moderate malocclusion in non-growing patients [11]. Another study demonstrated that Invisalign can be used for orthodontic tooth movements before and after orthognathic surgery without compromising the clinical outcome [12].

Dental bleaching is frequently requested by patients undergoing orthodontic treatment for aesthetic reasons [13]. It was reported that almost 90% of orthodontic patients requested dental bleaching during treatment [14]. Home bleaching is commonly used to whiten teeth among different bleaching methods due to its combined safety, treatment, and cost-effectiveness [15,16]. The American Dental Association recommends 10% carbamide peroxide as the gold-standard material for home bleaching [17,18].

A few studies have evaluated the effectiveness of dental bleaching during Invisalign orthodontic treatment [19,20,21]. However, only one study [19] evaluated the effect of dental bleaching on the microstructural properties and translucency of F22 aligners (Sweden & Martina SpA, Due Carrare, Italy). There is a lack of studies evaluating the effect of dental bleaching on the physical properties of Invisalign, including tensile strength, hardness, surface roughness, and translucency. Therefore, the aim of the present study was to evaluate the effect of 10% carbamide peroxide dental bleaching on the physical properties of Invisalign when the aligners were used as bleaching trays.

The null hypothesis was that 10% carbamide peroxide dental bleaching would have no effect on the physical properties (tensile strength, hardness, surface roughness, and translucency) of Invisalign.

## 2. Materials and Methods

### 2.1. Protocol

**Figure 1:**
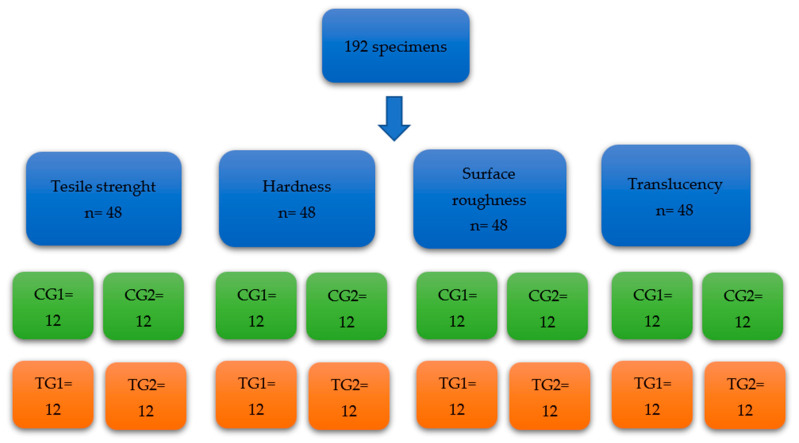
Flow diagram of specimen preparation.

In this in vitro study, twenty-two unused Invisalign aligners (Santa Clara, CA, USA) were used to prepare 144 specimens to test four physical properties: tensile strength, hardness, surface roughness, and translucency. To test each physical property, the specimens were allocated into four groups (Figure 1): a testing group at baseline (TG1, *n*= 12), a testing group after 2 weeks of dental bleaching (TG2, *n* = 12), a control group at baseline (CG1, *n* = 12), and a control group after 2 weeks of immersion in distilled water (CG2, *n* = 12). Specimens in CG1 and TG1 were immediately tested, while specimens in CG2 were stored in an incubator in distilled water at 37 °C for 2 weeks to simulate oral conditions. Next, specimens in TG2 were bleached using 10% carbamide peroxide (Opalescence PF 10%, Ultradent Products, New York City, NY, USA), which was applied on the internal surfaces of the specimens for 8 h a day at 37 °C, then wiped off; the specimens were kept in distilled water at mouth temperature in the incubator for 16 h. This process was repeated every day for 2 weeks before the specimens in TG2 were tested. This means that the 10% carbamide peroxide bleaching material was applied on the Invisalign specimens for a total of 112 h (8 h/day × 14 days) in TG2. The sample size was calculated according to other studies [19,20,21].

#### 2.1.1. Tensile Strength Testing

**Figure 2:**
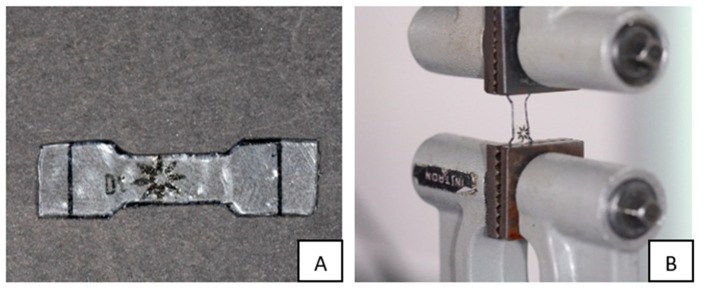
(**A**) A dumbbell-shaped specimen ready to be tested for tensile strength; note the extra length of the material to facilitate holding the specimen during the testing. (**B**) The dumbbell-shaped specimen in place ready for tensile strength testing.

Twelve unused Invisalign aligners (Santa Clara, CA, USA) were used to prepare 48 dumbbell-shaped specimens to test for tensile strength. The aligners were first heated using a Profom Vaccum forming machine (Profome Vacuum Former Machine III, Keystone, Burlington, NJ, USA), allowing for easier manipulation and flattening of the aligners. The aligners were then cut into four parts, and each part was used to create one sample, so four samples were prepared from each aligner. A resin jig printed (Prusa, MK3S+, Czech) with a dumbbell shape was used to mark the dumbbell shape on the aligner before cutting the four aligner parts into four identical specimens (Figure 2A). A universal testing machine (Instron 3369; Instron, Norwood, MA, USA) was used with a 500 N load cell to evaluate the point of failure of the specimens after the application of tensile force at a speed of 1 mm/min (Figure 2B).

#### 2.1.2. Hardness Testing

**Figure 3:**
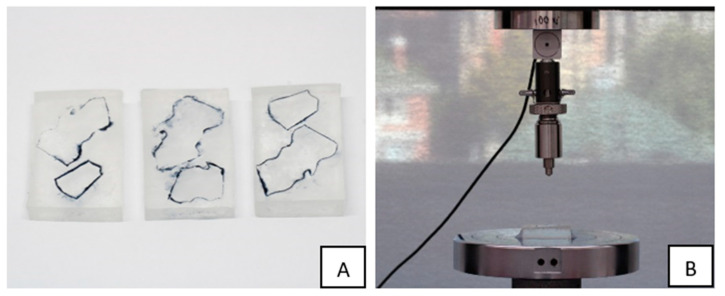
(**A**) The epoxy blocks after setting and polishing ready for hardness testing. (**B**) Each block was tested for hardness 12 times at 12 different sites using a universal testing machine (Instron 3369) at a load of 100 N and a loading time of 10 s at a speed of 1 mm/min. A diamond tip was used to create indentations, which were measured under a light microscope.

The remaining aligner material after preparing the dumbbell-shaped specimens was used to prepare specimens for hardness testing. First, condensation silicone (Protesil Labor, Vanini Dental, Florence, Italy) was used to make four moulds. Later, these moulds were used to create four epoxy resin blocks by pouring the epoxy resin (EpoFix, Struers, Ballerup, Denmark) into the moulds over the aligner specimens (Figure 3A). Next, a separator was used to paint the moulds to facilitate the removal of the epoxy resin out of the moulds after 24 h, which was the time required for the epoxy resin to set according to the manufacturer’s instructions. In addition, a marker was used to delineate the borders of the specimens to make it easier to visualize the specimens. After 24 h, the epoxy was sequentially polished with 500, 800, 1200, 2400, and 4000 grit silicon carbide paper (Struers, Ballerup, Denmark) coupled with a grinder polisher (model TegraPol-21, Struers, Ballerup, Denmark).

After polishing, each block was tested 12 times at 12 different sites using the universal testing machine (Instron 3369) at a load of 100 N and a loading time of 10 s (Figure 3B). Hardness testing included the creation of indentations by a diamond tip on the aligner specimens, after which the diameters of these indentations were measured under a light microscope (Nikon SMZ800N, Tokyo, Japan), and a formula (VHN = 1.854F/D^2^) * was used to calculate the Vickers hardness (VHN) using the average of these diameters for each specimen and eventually for each group (* where F is the applied load measured in kilograms-force, and D^2^ is the area of the indentation measured in square millimetres).

#### 2.1.3. Surface Roughness Testing

**Figure 4:**
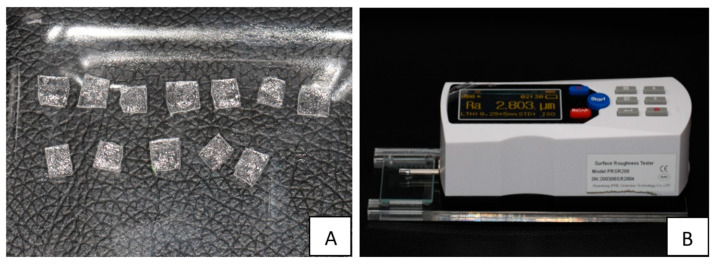
(**A**) Square-shaped specimens cut from the lingual surfaces of the mandibular incisors. (**B**) A surface roughness tester was used to test the surface roughness of the specimens.

Six mandibular aligners were used to prepare 24 square-shaped specimens (5 mm × 5 mm) cut from the lingual surfaces of the central and lateral incisors (Figure 4A). No attempts were made to heat the aligners for flattening in order to preserve the surface imprints for testing; instead, nearly flat areas were cut and tested directly for surface roughness using a surface roughness tester (Figure 4B). The specimens were also divided into four groups as mentioned in Section 2.1: CG1, CG2, TG1, and TG2. The only difference was that the same specimens were used to test surface roughness in the control group at baseline (CG1) and after 2 weeks of being stored in distilled water at 37 °C (CG2). Likewise, the same applied to the testing group, in which the same specimens were used to test the surface roughness at baseline (TG1) and after 2 weeks of bleaching using 10% carbamide peroxide (TG2). It is worth mentioning that the internal surfaces of the control and the testing groups where the patient would apply the bleaching agents were tested. Furthermore, the external surfaces of the specimens in the four groups were tested at baseline and after 2 weeks to check for any change that might affect the plaque affinity to Invisalign. Surface roughness was measured in Ra (roughness average) units, which is the most common unit used to calculate the surface roughness by measuring the average surface heights and depths across the surface.

#### 2.1.4. Translucency Testing

**Figure 5:**
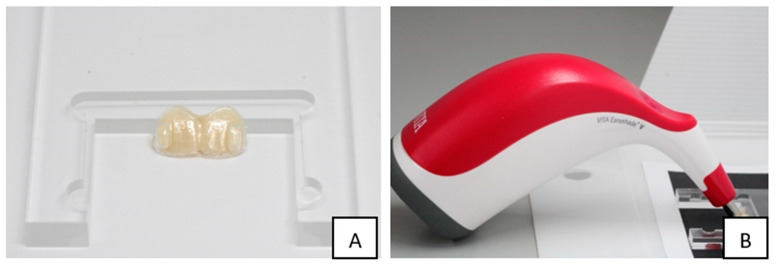
(**A**) Composite material (Filtek Supreme, 3M ESPE, MN, USA) was used to make a two-central-incisor mould, which was held against the specimen during translucency testing on a white and a black background. (**B**) Vita Easyshade*V was used to test the translucency; each specimen was tested twice: once with a white background and the other with a black background.

The two central incisors cut from four maxillary aligners were tested for translucency using a Vita Easyshade spectrophotometer (VITA Zahnfbarik, BadSackingen, Germany) (Figure 5A,B). Composite material (Filtek Supreme, 3M ESPE, Saint Paul, MN, USA) was used to create two central incisors using one of the aligners before cutting; this mould was held against the aligner specimens during testing. Each of two central incisors were tested six times at six different sites (disto-cervical, disto-incisal, mid-cervical, mid-incisal, mesio-cervical, and mesio-incisal), which accounted for six specimens. As per surface roughness testing, the same specimens in the control group were tested at baseline (CG1) and after storage in distilled water at 37 °C for 2 weeks (CG2). The specimens in the testing group were also tested at baseline (TG1) and after bleaching using 10% carbamide peroxide for 2 weeks (TG2). The translucency parameter was determined by calculating the colour difference between the specimen over a white (w) and a black (b) background using the following formula: TP = $\sqrt[{2}]{{\left(L_{w}-L_{b}\right)}^{2}+{\left(a_{w}-a_{b}\right)}^{2}+{\left(b_{w}-b_{b}\right)}^{2}}$, where a, b, and L are the colour vectors used in the CIE system and measured by the spectrophotometer.

### 2.2. Statistical Analysis

Statistical analysis was performed using SPSS v.27 for PC software (Statistical Package for Social Studies version 27, IBM, Chicago, IL, USA) to evaluate the effect of home bleaching using 10% carbamide peroxide bleaching material on the mechanical properties of Invisalign, with *p* < 0.05 considered statistically significant. Descriptive and bivariate analyses were conducted. Statistical tests for comparative analyses for samples in each group before and after treatment (immersion in distilled water or dental bleaching using 10% carbamide peroxide for 2 weeks) were conducted using a paired *t*-test and Wilcoxon signed rank test for normally distributed data and non-normally distributed data, respectively, which was decided by running a normality test (Shapiro–Wilk test). The statistical tests for comparative analyses for samples in CG2 and TG2 (after 2 weeks of immersion in distilled water or dental bleaching, respectively) were conducted using an independent sample *t*-test and Mann–Whitney test for normally distributed data and non-normally distributed data, respectively.

## 3. Results

### 3.1. Tensile Strength

**Figure 6:**
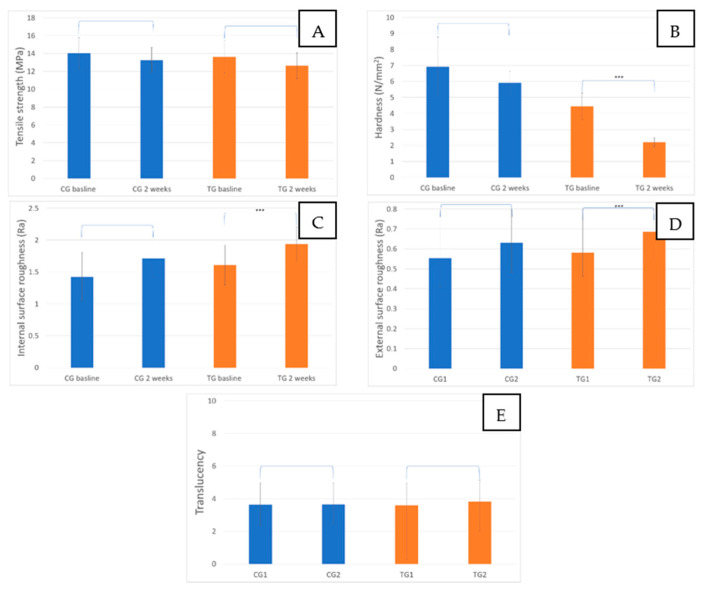
(**A**) Tensile strength of groups CG1, CG2, TG1, and TG2. No statistically significant difference was found between all groups. (**B**) Hardness of groups CG1, CG2, TG1, and TG2. A significant difference was found when comparing TG2 to TG1 and TG2 to CG2. (**C**) Internal surface roughness of groups CG1, CG2, TG1, and TG2. The only statistically significant difference (increase in surface roughness) was found when comparing TG2 to TG1. (**D**) External surface roughness of groups CG1, CG2, TG1, and TG2. A significant difference (increase) was found between TG2 and TG1. (**E**) Translucency values of groups CG1, CG2, TG1, and TG2. No statistically significant difference was found between all groups. Error bars represents standard deviation. *** represents *p*-value < 0.001.

Quantitative analysis showed that the tensile strength means for CG1 and CG2 were 14.04 ± 1.83 MPa and 13.26 ± 1.48 MPa, respectively. There was no statistically significant difference between the two groups (CG1 and CG2), with a *p*-value = 0.265. Evaluation of the effect of 10% carbamide peroxide dental bleaching on the tensile strength of Invisalign also showed no statistically significant difference between the mean values of groups TG1 and TG2, at 13.62 ± 1.9 MPa and 12.64 ± 1.5 MPa, respectively, with a *p*-value = 0.378 (Figure 6A). Furthermore, no statistically significant difference was found between groups CG2 and TG2 (*p*-value = 0.378) when comparing the effect of 2 weeks of immersion in distilled water to the effect of 2 weeks of dental bleaching at 37 °C on the tensile strength of Invisalign.

### 3.2. Hardness

Evaluation of the hardness in the control group at baseline (CG1) and after 2 weeks (CG2) revealed a reduction in the mean values from 6.93 ± 1.92 N/mm^2^ to 5.91 ± 0.78 N/mm^2^, respectively. However, the difference was not statistically significant, with a *p*-value = 0.101 (Figure 6B). Statistical analysis showed a significant difference between groups TG1 and TG2, with mean hardness values of 4.43 ± 0.86 N/mm^2^ and 2.2 ± 0.29 N/mm^2^, respectively, and a *p*-value < 0.001. A significant difference was found between groups CG2 and TG2, with a *p*-value < 0.001.

### 3.3. Surface Roughness

Evaluation of the internal surface roughness showed no statistically significant difference between groups CG1 and CG2, with mean values at 1.42 ± 0.39 Ra (average roughness) and 1.71 ± 0.3 Ra, respectively, and a *p*-value = 0.507 (Figure 6C). However, the internal surface roughness was found to be increased after the application of 10% carbamide peroxide for 2 weeks, from 1.6 ± 0.32 Ra (TG1) to 1.93 ± 0.28 Ra (TG2). Furthermore, statistical analysis showed a significant difference between the two groups (TG1 and TG2) with a *p*-value = 0.007. Groups CG2 and TG2 were found to be not statistically different, with a *p*-value = 0.068.

Evaluation of the external surface roughness showed no statistically significant difference between groups CG1 and CG2, with mean values of 0.55 ± 0.14 Ra and 0.63 ± 0.15 Ra, respectively, and a *p*-value = 0.230 (Figure 6D). However, there was a statistically significant difference between groups TG1 and TG2, as evidenced by the increase in external surface roughness, mean values increasing from 0.58 ± 0.12 Ra to 0.68 ± 0.13 Ra after 2 weeks of dental bleaching and a *p*-value < 0.001.

### 3.4. Translucency

Translucency testing showed maintenance of values after 2 weeks of immersion in distilled water at 37 °C, while applying 10% carbamide peroxide for 2 weeks resulted in a slight increase in the translucency values when TG2 was compared to TG1. However, no statistically significant difference was found when comparing group CG2 to CG1, TG2 to TG1, and TG2 to CG2. The mean values of translucency were reported for groups CG1, CG2, TG1, and TG2 at 3.64 ± 1.39, 3.64 ± 1.22, 3.60 ± 1.53, and 3.8 ± 1.89, respectively (Figure 6E). A summary of the data resulting from testing the four different physical properties is presented in Table 1.

## 4. Discussion

Orthodontic treatment using clear aligners, including Invisalign, is becoming increasingly popular for aesthetic reasons [1,22]. Aesthetics are also the driving force for patients requesting dental bleaching during orthodontic treatment [13]. Furthermore, as studies have shown no superiority of reservoir trays over non-reservoir trays, it is now possible to use Invisalign as a bleaching tray to accommodate the bleaching material at night to combine orthodontic treatment with dental bleaching [23]. This treatment modality could eliminate the need for another set of impressions, further decreasing the total cost and time [19,20,21].

Some studies have suggested delaying dental bleaching to the later stages of orthodontic treatment to prevent the effect of the presence of composite attachments on the final bleaching result, while other studies have suggested the use of lingual attachments instead of labial attachments to ensure homogenous tooth whitening [19,20]. However, it was reported that the small molecular size of the bleaching material enabled the penetration of the bleaching material molecules into dentine adjacent to the attachments and resulted in the bleaching of these areas without the need for direct contact between the bleaching material and the tooth surface [13]. Therefore, dental bleaching can be applied concurrently with Invisalign orthodontic treatment. However, it might be more appropriate to delay dental bleaching to later stages when teeth are more aligned with severe crowding.

Several studies have evaluated the effect of dental bleaching on enamel microhardness [24,24], showing a controversial effect of dental bleaching on enamel microhardness. In addition, some studies have reported a negative impact of dental bleaching on enamel microhardness and roughness [24,25]. However, it has been reported that lower-concentration bleaching agents are associated with fewer adverse effects on enamel surface microstructural properties than the adverse effects resulting from higher-concentration bleaching agents [26,27]. Moreover, a systematic review [28] reported no significant changes in enamel microhardness after bleaching with 10% dental carbamide peroxide over 7, 14, and 21 days.

A number of studies have been conducted to evaluate the effectiveness of dental bleaching during orthodontic treatment using clear aligners [19,20,21]. Oliverio et al. (2019) reported shade improvement when F22 clear aligners were used as bleaching trays at night for dental bleaching. The authors showed that the highest concentration of carbamide peroxide (16%) applied for 14 days was associated with better shade improvement compared to a lower concentration of bleaching agent or the same concentration applied for 7 days. Furthermore, the authors reported no change in the microstructural properties or the translucency of the aligners when evaluated under a scanning electron microscope (SEM) and a double-beam spectrophotometer [19]. Levrini et al. (2020) reported no significant difference in final tooth shade when using Invisalign as bleaching trays compared to reservoir bleaching trays using 10% carbamide peroxide after 10 days [20]. Furthermore, Seleem et al. (2021) showed that dental bleaching using 10% carbamide peroxide using Invisalign aligners as bleaching trays was clinically effective and resulted in tooth shade improvement, with an average tooth shade improvement of 3.5 shades when evaluated using the Vita classic shade guide after 2 weeks [21].

Moreover, some studies have evaluated the effect of intraoral ageing on the physical properties of Invisalign. Therefore, evaluation of the tensile strength of Invisalign is highly important, as sufficient stiffness is required for force delivery. Two studies reported a reduction in the tensile strength of Invisalign when comparing unused Invisalign to used Invisalign aligners after 2 weeks [29,30]. However, Fang et al. (2020) reported no significant changes in tensile strength, with mean values of 842 ± 63 and 806 ± 19 before and after intraoral use by patients, respectively [31]. The difference in the results between the three mentioned studies [29,30,31] is mainly attributed to the changes in the properties of the Invisalign material and the testing duration. The findings of the present study are in agreement with those reported by Fang et al. (2020), with no significant change in the tensile strength of Invisalign aligners after immersion in distilled water or following the application of 10% carbamide peroxide for 2 weeks. These findings can be attributed to the new material (Smart Track LD30) currently used for the fabrication of Invisalign aligners, which has been found to have a higher elasticity and superior mechanical properties compared to the former material (Exceed EX30) [31].

Moreover, in the present study, the tensile strength was tested using a universal testing machine (Instron 3369; Instron, Norwood, MA, USA), which is known for its accuracy compared to the indentation test that was used in a previous study [29]. Furthermore, the duration that was used in this study was 14 days to mimic the clinical situation of at-home bleaching, which is less than the duration (29 days) that was used in one of the studies that showed a reduction in tensile strength [29]. Therefore, according to these results, it is anticipated that 10% carbamide peroxide home bleaching using Invisalign aligner as a bleaching tray will not affect the force delivery of Invisalign and the clinical treatment outcome.

Another physical property of Invisalign evaluated in the literature is hardness. Previous studies have suggested that a reduction in Invisalign hardness would decrease the wear resistance of Invisalign and, in contrast, an increase in hardness would result in increased patient discomfort and difficulty in the insertion and removal of Invisalign [30,32]. One study reported a reduction in Invisalign hardness after 2 weeks of use [29]. Another study reported an opposite finding [30]. A reduction in Invisalign hardness was reported in one study [29] as the result of the wear of Invisalign, which happened whenever Invisalign had contact with enamel and the attachments, which had higher hardness compared to that of Invisalign. This study measured Vickers hardness by creating indentations on the Invisalign specimens using a diamond tip fixed on a universal testing machine (Instron 3369; Instron, Norwood, MA, USA); then, the dimensions of these indentations were measured under a light microscope. Although it was argued that there are some limitations to the measurement of Vickers hardness, including the resolution of the optical system and the perception of the operator and it has been suggested that the Martens hardness test is preferable [32], another study reported that there were no differences in measurement between Vickers and Martens hardness testing when both were used for the measurement of dental material hardness [33]. The findings of the present study showed no significant changes in the hardness of Invisalign aligners after 2 weeks of immersion in distilled water. On the other hand, the present study showed a reduction in the hardness of Invisalign aligners after 2 weeks of application of 10% carbamide peroxide. The reduction in hardness of Invisalign material could be linked to a possible chemical interaction between the bleaching material and the Invisalign aligner material, as a previous study showed that the application of certain types of cleansers was associated with changes in the mechanical properties of clear aligners [34]. Furthermore, this decrease in Invisalign hardness and, in turn, the decrease in wear resistance is not anticipated to create a problem, as each set of Invisalign is worn for 2 weeks only.

Papadopoulou et al. (2019) evaluated the surface roughness of the internal surfaces of used (for 1 week and 2 weeks) and unused Invisalign aligners [35]. The authors reported a reduction in surface roughness after 1 week and 2 weeks of intraoral use attributed to the wear of Invisalign aligners as a result of contacting a harder substance, i.e., enamel or composite resin for attachments [35]. There was no significant difference in surface roughness between Invisalign aligners used for 1 week and those used for 2 weeks [35]. Furthermore, the authors explained that this decrease in surface roughness and material coefficient of friction resulted in the deterioration of Invisalign aligner retention after intraoral use and in the lower plaque affinity of the aligners [35]. A surface roughness tester was used in the present study due to availability and familiarity with the machine. The present study showed no significant differences in external and internal surface roughness after immersion in distilled water for 2 weeks. Moreover, a statistically significant increase in the roughness of the external and internal surfaces of Invisalign aligners was found after the application of 10% carbamide peroxide for 2 weeks. The increased surface roughness of Invisalign after the application of 10% carbamide peroxide can be further linked to the reduction in the Invisalign hardness, which is anticipated to decrease the wear resistance and, in turn, to increase the surface roughness of Invisalign. The increase in the external surface roughness can be anticipated to be associated with increased plaque affinity, which emphasizes the importance of oral hygiene instructions and reinforcement by the dentist and the importance of oral hygiene maintenance by the patient.

No studies in the literature have assessed the translucency of Invisalign after intra-oral use. Oliverio et al. (2019) reported maintenance in the transparency of F22 aligners after 2 weeks of dental bleaching using different concentrations of hydrogen and carbamide peroxide [19]. A spectrophotometer was used in this study to measure the translucency of Invisalign, as previous studies showed its accuracy and reliability [19,36]. The present study also showed maintenance of Invisalign translucency after application of 10% carbamide peroxide for 2 weeks. This maintenance of Invisalign translucency can be attributed to the chemical stability of the new material used for the fabrication of Invisalign Smart Track LD30. The results of this study show that the appearance of Invisalign is not inversely affected when used as a bleaching tray at night. However, future clinical trials further investigating any change in the colour and translucency of Invisalign and evaluating patient satisfaction are needed.

To the best of our knowledge, no studies have been conducted to evaluate the effect of using 10% carbamide peroxide on the physical properties of Invisalign when used as a bleaching tray at night. Therefore, the aim of the present in vitro study was to investigate the effect of 10% carbamide peroxide on the tensile strength, hardness, surface roughness, and translucency of Invisalign. In this study, 10% carbamide peroxide was applied for 8 h, as mentioned previously, to mimic the application of bleaching material overnight; however, a small number of studies [36,37,38] showed that the application of 10% carbamide peroxide for 2 h or a maximum of 4 h was efficient in whitening teeth and providing a good aesthetic outcome. This can be used in evaluating the effect of 10% carbamide peroxide when applied for 2 h on the physical properties, i.e., the surface roughness as and the hardness of Invisalign, in future research. The results of this study showed no significant changes in tensile strength before and after the application of 10% carbamide peroxide for 2 weeks, with mean values of 13.62 ± 1.9 MPa and 12.64 ± 1.5 MPa, respectively. Furthermore, translucency showed maintenance of values, with no statistically significant difference before and after application of 10% carbamide peroxide for 2 weeks, with mean values of 3.60 ± 1.53 and 3.8 ± 1.89, respectively. However, the results showed a reduction in Invisalign hardness after 2 weeks of application of 10% carbamide peroxide bleaching material from 4.43 ± 0.86 N/mm^2^ to 2.2 ± 0.29 N/mm^2^, while the external and internal surface roughness was increased from 0.58 ± 0.12 Ra to 0.68 ± 0.13 Ra and from 1.6 ± 0.32 Ra to 1.93 ± 0.28 Ra, respectively.

## 5. Conclusions

The present study showed that Invisalign can be used for dental bleaching without excessive distortion or degradation of the aligner material. However, future clinical trials are required to further assess the feasibility of using Invisalign for dental bleaching. Such studies can also be used to assess the efficiency of dental bleaching and Invisalign orthodontic treatment when the two treatments are combined.

## Figures and Tables

**Table 1:** The mean confidence interval and standard deviation of the tested specimens for the four different physical properties: tensile strength, hardness, surface roughness, and translucency.

**Testing**	**Tensile Strength CG1**	**Tensile Strength CG2**	**Tensile Strength TG1**	**Tensile Strength TG2**
Mean (±S.D *)	14.04 ± 1.83	13.26 ± 1.48	13.62 ± 1.9	12.64 ± 1.5
Median	14.14	13.28	12.89	12.59
95% Confidence interval (LB, UB)	12.87, 15.20	12.31, 14.20	12.41, 14.83	11.68, 13.59
**Testing**	**Hardness CG1**	**Hardness CG2**	**Hardness TG1**	**Hardness TG2**
Mean (±S.D)	6.93 ± 1.92	5.91 ± 0.78	4.43 ± 0.86	2.2 ± 0.29
Median	6.13	5.8	4.45	2.13
95% Confidence interval (LB, UB)	5.70, 8.15	5.41, 6.41	3.88, 4.99	2.01, 2.38
**Testing**	**Surface Roughness CG1**	**Surface Roughness CG2**	**Surface Roughness TG1**	**Surface Roughness TG2**
Mean (±S.D)	1.42 ± 0.39	1.71 ± 0.3	1.6 ± 0.32	1.93 ± 0.28
Median	1.45	1.67	1.7	1.93
95% Confidence interval (LB, UB)	1.16, 1.67	1.52. 1.90	1.39. 1.81	1.75, 2.11
**Testing (External Surface)**	**Surface Roughness CG1**	**Surface Roughness CG2**	**Surface Roughness TG1**	**Surface Roughness TG2**
Mean (±S.D)	0.55 ± 0.14	0.63 ± 0.15	0.58 ± 0.12	0.68 ± 0.13
Median	0.51	0.57	0.55	0.65
95% Confidence interval (LB, UB)	0.45, 0.64	0.53, 0.72	0.50, 0.65	0.59, 0.77
**Testing**	**Translucency CG1**	**Translucency CG2**	**Translucency TG1**	**Translucency TG2**
Mean (±S.D)	3.64 ± 1.39	3.64 ± 1.22	3.60 ± 1.53	3.8 ± 1.89
Median	3.62	3.55	3.71	3.78
95% Confidence interval (LB, UB)	2.75, 4.52	2.87, 4.42	2.62, 4.57	2.60, 5.00

* S.D: Standard deviation.

## Data Availability

The data presented in this study are available upon request from the corresponding author.

## References

[1] Putrino A., Barbato E., Galluccio G. (2021). Clear Aligners: Between evolution and efficiency—A scoping review. Int. J. Environ. Res. Public Health.

[2] Tartaglia G.M., Mapelli A., Maspero C., Santaniello T., Serafin M., Farronato M., Caprioglio A. (2021). Direct 3D printing of clear orthodontic aligners: Current state and future possibilities. Materials.

[3] Rossini G., Parrini S., Castroflorio T., Deregibus A., Debernardi C.L. (2015). Efficacy of clear aligners in controlling orthodontic tooth movement: A systematic review. Angle Orthod..

[4] Weir T. (2017). Clear aligners in orthodontic treatment. Aust. Dent. J..

[5] Wheeler T.T. (2017). Orthodontic clear aligner treatment. Semin. Orthod..

[6] Kuncio D.A. (2014). Invisalign: Current guidelines for effective treatment. N. Y. State Dent. J..

[7] Borda A.F., Garfinkle J.S., Covell D.A., Wang M., Doyle L., Sedgley C.M. (2020). Outcome assessment of orthodontic clear aligner vs fixed appliance treatment in a teenage population with mild malocclusions. Angle Orthod..

[8] Miller K.B., McGorray S.P., Womack R., Quintero J.C., Perelmuter M., Gibson J., Dolan T.A., Wheeler T.T. (2007). A comparison of treatment impacts between Invisalign aligner and fixed appliance therapy during the first week of treatment. Am. J. Orthod. Dentofac. Orthop..

[9] Pacheco-Pereira C., Brandelli J., Flores-Mir C. (2018). Patient satisfaction and quality of life changes after Invisalign treatment. Am. J. Orthod. Dentofac. Orthop..

[10] Alajmi S., Shaban A., Al-Azemi R. (2020). Comparison of Short-Term Oral Impacts Experienced by Patients Treated with Invisalign or Conventional Fixed Orthodontic Appliances. Med. Princ. Pract..

[11] Papadimitriou A., Mousoulea S., Gkantidis N., Kloukos D. (2018). Clinical effectiveness of invisalign^®^ orthodontic treatment: A systematic review. Prog. Orthod..

[12] Kankam H., Madari S., Sawh-Martinez R., Bruckman K.C., Steinbacher D.M. (2019). Comparing outcomes in orthognathic surgery using clear aligners versus conventional fixed appliances. J. Craniofacial Surg..

[13] Sword R.J., Haywood V.B. (2020). Teeth bleaching efficacy during clear aligner orthodontic treatment. Compend. Contin. Educ. Dent..

[14] Slack M.E., Swift E.J., Rossouw P.E., Phillips C. (2013). Tooth whitening in the orthodontic practice: A survey of orthodontists. Am. J. Orthod. Dentofac. Orthop..

[15] Haywood V.B., Heymann H.O. (1989). Nightguard vital bleaching. Quintessence Int..

[16] Mounika A., Mandava J., Roopesh B., Karri G. (2018). Clinical evaluation of color change and tooth sensitivity with in-office and home bleaching treatments. Indian J. Dent. Res..

[17] Chemin K., Rezende M., Milan F.M., Dantas T.B., Gomes K.D.N., Kossatz S. (2018). Clinical evaluation of 10% hydrogen peroxide on tooth sensitivity and effectiveness in at home dental bleaching. J. Contemp. Dent. Pract..

[18] Sutil E., da Silva K.L., Terra R.M.O., Burey A., Rezende M., Reis A., Loguercio A.D. (2022). Effectiveness and adverse effects of at-home dental bleaching with 37% versus 10% carbamide peroxide: A randomized, blind clinical trial. J. Esthet. Restor. Dent..

[19] Oliverio T., Cremonini F., Lombardo L., Siciliani G. (2019). Tooth whitening in association with clear aligner treatment. J. Clin. Orthod..

[20] Levrini L., Paracchini L., Bakaj R., Diaconu A., Cortese S. (2020). Dental bleaching during orthodontic treatment with aligners. Int. J. Esthet. Dent..

[21] Seleem D., Dadjoo S., Ha A., Santos C., Mirfarsi S., Matsumura-Lem K., Lazarchik D. (2021). Effect of 10% carbamide peroxide on tooth shade, plaque index and gingival index during invisalign treatment. Dent. J..

[22] Dos Santos P.R., Meneghim M.C., Ambrosano G.M.B., Filho M.V., Vedovello S.A.S. (2017). Influence of quality of life, self-perception, and self-esteem on orthodontic treatment need. Am. J. Orthod. Dentofac. Orthop..

[23] Martina S., Rongo R., Bucci R., Razionale A.V., Valletta R., D’Antò V. (2019). In vitro cytotoxicity of different thermoplastic materials for clear aligners. Angle Orthod..

[24] Basting R., Júnior A.L.R., Serra M.C. (2001). The effect of 10% carbamide peroxide bleaching material on microhardness of sound and demineralized enamel and dentin in situ. Oper Dent..

[25] Lopes G.C., Bonissoni L., Baratieri L.N., Vieira L.C.C., Monteiro S. (2002). Effect of bleaching agents on the hardness and morphology of enamel. J. Esthet. Restor. Dent..

[26] Furlan I.S., Bridi E.C., Amaral F.L.B.D., França F.M.G., Turssi C.P., Basting R. (2017). Effect of high- or low-concentration bleaching agents containing calcium and/or fluoride on enamel microhardness. Gen. Dent..

[27] Karimi Z., Saoui H., Sakout M., Abdallaoui F. (2021). Effect of Vital Bleaching on Micromorphology of Enamel Surface: An in Vitro Study. Prim. Dent. J..

[28] Zanolla J., Marques A., Costa D., Souza A., Coutinho M. (2017). Influence of tooth bleaching on dental enamel microhardness: A systematic review and meta-analysis. Aust. Dent. J..

[29] Bradley T.G., Teske L., Eliades G., Zinelis S., Eliades T. (2016). Do the mechanical and chemical properties of Invisalign^TM^ appliances change after use? A retrieval analysis. Eur. J. Orthod..

[30] Condo R., Pazzini L., Cerroni L., Pasquantonio G., Lagana G., Pecora A., Mussi V., Rinaldi A., Mecheri B., Licoccia S. (2018). Mechanical properties of “two generations” of teeth aligners: Change analysis during oral permanence. Dent. Mater J..

[31] Fang D., Li F., Zhang Y., Bai Y., Wu B.M. (2020). Changes in mechanical properties, surface morphology, structure, and composition of Invisalign material in the oral environment. Am. J. Orthod. Dentofac. Orthop..

[32] Westrich R.M. (1985). Use of the Scanning Electron Microscope in Microhardness Testing of High-Hardness Materials.

[33] Shahdad S.A., McCabe J.F., Bull S., Rusby S., Wassell R.W. (2007). Hardness measured with traditional Vickers and Martens hardness methods. Dent. Mater..

[34] Iliadi A., Enzler V., Polychronis G., Peltomaki T., Zinelis S., Eliades T. (2022). Effect of cleansers on the composition and mechanical properties of orthodontic aligners in vitro. Prog. Orthod..

[35] Papadopoulou A.K., Cantele A., Polychronis G., Zinelis S., Eliades T. (2019). Changes in Roughness and Mechanical Properties of Invisalign^®^ Appliances after One- and Two-Weeks Use. Materials.

[36] Daniele V., Macera L., Taglieri G., Spera L., Marzo G., Quinzi V. (2021). Color Stability, Chemico-Physical and Optical Features of the Most Common PETG and PU Based Orthodontic Aligners for Clear Aligner Therapy. Polymers.

[37] Matis B.A., Cochran M.A., Eckert G. (2009). Review of the effectiveness of various tooth whitening systems. Oper Dent..

[38] Bernardon J.K., Ferrari P., Baratieri L.N., Rauber G.B. (2015). Comparison of treatment time versus patient satisfaction in at-home and in-office tooth bleaching therapy. J. Prosthet. Dent..

